# Overweight BMI compromises survival and correlates with aggressive phenotypes in early-onset but not late-onset rectal cancer patients

**DOI:** 10.1093/oncolo/oyag380

**Published:** 2026-09-25

**Authors:** Jinlin Cai, Tuoyang Li, Jingrong Weng, Ziye Zhang, Junyi Han, Ziming Li, Zhihao Xu, Ying Du, Jinxin Lin, Meijin Huang, Xiaolin Wang, Yanxin Luo, Huichuan Yu

**Affiliations:** Department of General Surgery (Colorectal Surgery), The Sixth Affiliated Hospital, Sun Yat-sen University, Guangzhou, Guangdong, 510655, China; Guangdong Institute of Gastroenterology, Guangzhou, Guangdong, 510655, China; Guangdong Provincial Key Laboratory of Colorectal and Pelvic Floor Diseases, The Sixth Affiliated Hospital, Sun Yat-sen University, Guangzhou, Guangdong, China; Biomedical Innovation Center, The Sixth Affiliated Hospital, Sun Yat-sen University, Guangzhou, Guangdong, 510655, China; Department of General Surgery (Colorectal Surgery), The Sixth Affiliated Hospital, Sun Yat-sen University, Guangzhou, Guangdong, 510655, China; Guangdong Institute of Gastroenterology, Guangzhou, Guangdong, 510655, China; Guangdong Provincial Key Laboratory of Colorectal and Pelvic Floor Diseases, The Sixth Affiliated Hospital, Sun Yat-sen University, Guangzhou, Guangdong, China; Biomedical Innovation Center, The Sixth Affiliated Hospital, Sun Yat-sen University, Guangzhou, Guangdong, 510655, China; Key Laboratory of Human Microbiome and Chronic Diseases (Sun Yat-sen University), Ministry of Education, Guangzhou, Guangdong, 510655, China; Department of General Surgery (Colorectal Surgery), The Sixth Affiliated Hospital, Sun Yat-sen University, Guangzhou, Guangdong, 510655, China; Guangdong Institute of Gastroenterology, Guangzhou, Guangdong, 510655, China; Guangdong Provincial Key Laboratory of Colorectal and Pelvic Floor Diseases, The Sixth Affiliated Hospital, Sun Yat-sen University, Guangzhou, Guangdong, China; Biomedical Innovation Center, The Sixth Affiliated Hospital, Sun Yat-sen University, Guangzhou, Guangdong, 510655, China; Key Laboratory of Human Microbiome and Chronic Diseases (Sun Yat-sen University), Ministry of Education, Guangzhou, Guangdong, 510655, China; Department of General Surgery (Colorectal Surgery), The Sixth Affiliated Hospital, Sun Yat-sen University, Guangzhou, Guangdong, 510655, China; Guangdong Institute of Gastroenterology, Guangzhou, Guangdong, 510655, China; Guangdong Provincial Key Laboratory of Colorectal and Pelvic Floor Diseases, The Sixth Affiliated Hospital, Sun Yat-sen University, Guangzhou, Guangdong, China; Biomedical Innovation Center, The Sixth Affiliated Hospital, Sun Yat-sen University, Guangzhou, Guangdong, 510655, China; Department of General Surgery (Colorectal Surgery), The Sixth Affiliated Hospital, Sun Yat-sen University, Guangzhou, Guangdong, 510655, China; Guangdong Institute of Gastroenterology, Guangzhou, Guangdong, 510655, China; Guangdong Provincial Key Laboratory of Colorectal and Pelvic Floor Diseases, The Sixth Affiliated Hospital, Sun Yat-sen University, Guangzhou, Guangdong, China; Guangdong Institute of Gastroenterology, Guangzhou, Guangdong, 510655, China; Guangdong Provincial Key Laboratory of Colorectal and Pelvic Floor Diseases, The Sixth Affiliated Hospital, Sun Yat-sen University, Guangzhou, Guangdong, China; Biomedical Innovation Center, The Sixth Affiliated Hospital, Sun Yat-sen University, Guangzhou, Guangdong, 510655, China; Department of General Surgery (Colorectal Surgery), The Sixth Affiliated Hospital, Sun Yat-sen University, Guangzhou, Guangdong, 510655, China; Guangdong Institute of Gastroenterology, Guangzhou, Guangdong, 510655, China; Guangdong Provincial Key Laboratory of Colorectal and Pelvic Floor Diseases, The Sixth Affiliated Hospital, Sun Yat-sen University, Guangzhou, Guangdong, China; Biomedical Innovation Center, The Sixth Affiliated Hospital, Sun Yat-sen University, Guangzhou, Guangdong, 510655, China; Department of General Surgery (Colorectal Surgery), The Sixth Affiliated Hospital, Sun Yat-sen University, Guangzhou, Guangdong, 510655, China; Guangdong Institute of Gastroenterology, Guangzhou, Guangdong, 510655, China; Guangdong Provincial Key Laboratory of Colorectal and Pelvic Floor Diseases, The Sixth Affiliated Hospital, Sun Yat-sen University, Guangzhou, Guangdong, China; Biomedical Innovation Center, The Sixth Affiliated Hospital, Sun Yat-sen University, Guangzhou, Guangdong, 510655, China; Department of General Surgery (Colorectal Surgery), The Sixth Affiliated Hospital, Sun Yat-sen University, Guangzhou, Guangdong, 510655, China; Guangdong Institute of Gastroenterology, Guangzhou, Guangdong, 510655, China; Guangdong Provincial Key Laboratory of Colorectal and Pelvic Floor Diseases, The Sixth Affiliated Hospital, Sun Yat-sen University, Guangzhou, Guangdong, China; Guangdong Institute of Gastroenterology, Guangzhou, Guangdong, 510655, China; Guangdong Provincial Key Laboratory of Colorectal and Pelvic Floor Diseases, The Sixth Affiliated Hospital, Sun Yat-sen University, Guangzhou, Guangdong, China; Guangdong Institute of Gastroenterology, Guangzhou, Guangdong, 510655, China; Guangdong Provincial Key Laboratory of Colorectal and Pelvic Floor Diseases, The Sixth Affiliated Hospital, Sun Yat-sen University, Guangzhou, Guangdong, China; Biomedical Innovation Center, The Sixth Affiliated Hospital, Sun Yat-sen University, Guangzhou, Guangdong, 510655, China; Department of General Surgery (Colorectal Surgery), The Sixth Affiliated Hospital, Sun Yat-sen University, Guangzhou, Guangdong, 510655, China; Guangdong Institute of Gastroenterology, Guangzhou, Guangdong, 510655, China; Guangdong Provincial Key Laboratory of Colorectal and Pelvic Floor Diseases, The Sixth Affiliated Hospital, Sun Yat-sen University, Guangzhou, Guangdong, China; Biomedical Innovation Center, The Sixth Affiliated Hospital, Sun Yat-sen University, Guangzhou, Guangdong, 510655, China; Sun Yat-sen University Institute of Advanced Studies Hong Kong, Hong Kong, China; Guangdong Institute of Gastroenterology, Guangzhou, Guangdong, 510655, China; Guangdong Provincial Key Laboratory of Colorectal and Pelvic Floor Diseases, The Sixth Affiliated Hospital, Sun Yat-sen University, Guangzhou, Guangdong, China; Biomedical Innovation Center, The Sixth Affiliated Hospital, Sun Yat-sen University, Guangzhou, Guangdong, 510655, China; Key Laboratory of Human Microbiome and Chronic Diseases (Sun Yat-sen University), Ministry of Education, Guangzhou, Guangdong, 510655, China

**Keywords:** early-onset rectal cancer, obesity, survival outcome, aggressive phenotype

## Abstract

**Background:**

The incidence of early-onset colorectal cancer (EOCRC) is rising globally, presenting unique clinical challenges due to its aggressive nature compared to late-onset cases. While obesity is a known risk factor for carcinogenesis, its prognostic significance specifically on the EOCRC population remains underexplored. We hypothesized that the association of metabolic dysregulation with tumor aggressiveness and survival may depend on the age of onset.

**Methods:**

A total of 324 patients with stage II/III rectal cancer from the FOWARC trial were stratified by BMI (using the Asian overweight cut-off of 24 kg/m^2^) and age of onset (early-onset <50 years vs. late-onset ≥50 years). The clinicopathologic characteristics, treatment response, complications, and survival outcomes were compared.

**Results:**

The prognostic profile was heterogeneous between patients with high and low BMI. Notably, age had opposing prognostic value in the two groups. In the early-onset subgroups, patients with high BMI exhibited more prevalent aggressive phenotypes, including T4 stage, MRF+, elevated tumor length/thickness, and pretreatment CEA. However, in the late-onset subgroups, those phenotypes were less in the patients with high BMI. For short-term outcome, the incidence of pathological complete response after neoadjuvant treatment and complications was similar between two groups. In the early-onset subgroups, the overall (*P *= .003) and disease-free (*P *= .001) survival outcomes were significantly worse in the patients with high BMI compared to those with low BMI. However, this difference was not observed in the late-onset subgroups.

**Conclusion:**

The association of overweight and obesity with aggressive tumor phenotypes and poor survival outcomes is specifically observed in early-onset rectal cancer, which highlights the need for BMI-specific therapeutic strategies and weight management interventions in these vulnerable cases.

## Graphical abstract

**Graphical Abstract:**
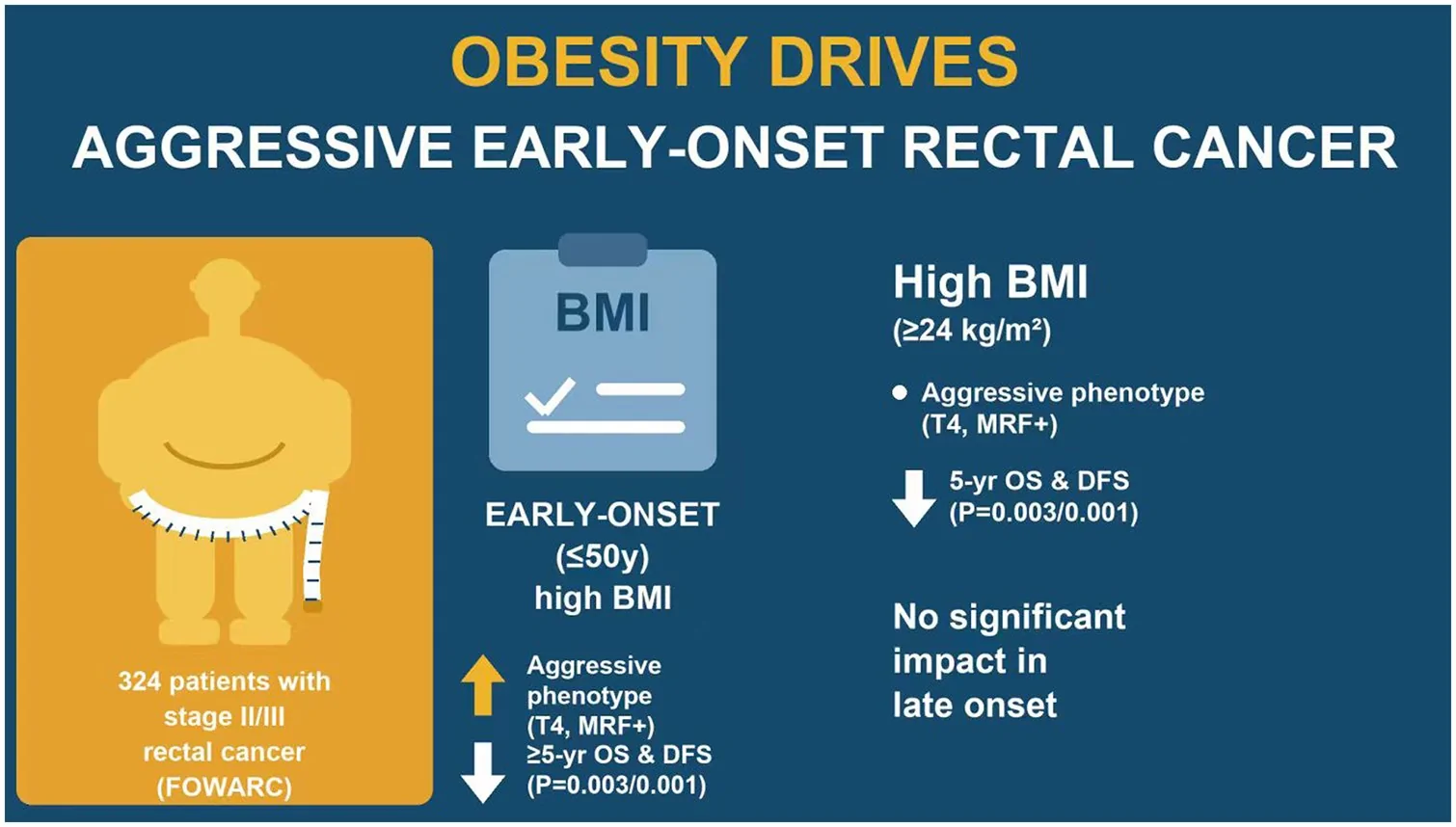

Implications for practice This study reveals that elevated BMI significantly worsens survival and leads to more aggressive tumors specifically in younger patients—a trend not observed in older populations. These findings suggest that younger patients are uniquely vulnerable to the biological effects of excess weight. Clinically, this highlights the urgent need for personalized treatment plans and weight management interventions tailored for young patients to improve their long-term health and survival outcomes.

## Introduction

Colorectal cancer (CRC) has been traditionally viewed as a disease of the elderly. However, the global incidence of early-onset colorectal cancer (EOCRC) that is diagnosed younger than 50 years has risen alarmingly over the past two decades, with rectal cancer demonstrating a significant increase among gastrointestinal malignancies.^1^^,^^2^ Unlike late-onset CRC (LOCRC), EOCRC frequently presents with advanced stages and aggressive pathological features, posing a significant challenge to current age-agnostic treatment paradigms.^2^^,^^3^ Despite aggressive multimodal therapies, EOCRC patients exhibit a disproportionately high risk of recurrence, underscoring the urgent need to identify specific risk stratification and tailored therapeutic strategies.^4^^,^^5^

This epidemiological shift coincides with an obesity pandemic, where increasing adults aged 20-49 now meet criteria for overweight and obesity worldwide.^6^ Emerging evidence suggests these dual trends may be pathologically intertwined, and the link between obesity and overall CRC risk is clear.^7^^,^^8^ The mechanisms of this link involve chronic inflammation, insulin resistance, and adipokine dysregulation.^9^^,^^10^ However, the prognostic value of body mass index (BMI) in established rectal cancer remains controversial, often described as the obesity paradox, where overweight or obesity status may seemingly protect against cancer cachexia in older populations and prime immune cells in cancer immunotherapy.^11–15^ This paradox is further complicated by the growing recognition of sarcopenic obesity, the coexistence of excess adiposity with low skeletal muscle mass, a condition known to be a poor prognostic factor that is often masked by a normal or elevated BMI.^16^^,^^17^

We hypothesized that the obesity paradox is not a universal phenomenon but is instead dependent on age, and that this interaction masks the true detrimental effects of metabolic dysregulation in younger patients. In the context of EOCRC, where the immune system is typically more robust and sarcopenia is less prevalent than in older patients undergoing immunosenescence, we posited that elevated BMI would serve as a more direct measure of obesity-induced metabolic stress. Consequently, its pro-tumorigenic effects would be unmasked, acting as a potent driver of tumor progression.

To test this hypothesis, this study leverages high-quality prospective data from the FOWARC trial, a multi-center trial that compared neoadjuvant mFOLFOX6 (modified infusional fluorouracil, leucovorin, and oxaliplatin) with or without radiation versus standard fluorouracil-based chemoradiotherapy in patients with locally advanced rectal cancer,^18^ to investigate the specific impact of overweight and obesity on the clinical trajectory of early-onset versus late-onset rectal cancer. By dissecting this age-BMI interaction, we aim to provide evidence for precision risk stratification and inform weight-management strategies in the clinical management of EOCRC.

## Methods

### Patient cohort

A total of 324 patients with stage II/III rectal cancer from the FOWARC trial were selected and analyzed in this study. In the FOWARC trial, a randomization approach was adopted to allocate patients into three treatment arms at a ratio of 1:1:1.^18^ Among them, two arms involved neoadjuvant radiation treatment (fluorouracil or mFOLFOX6 plus radiotherapy). The remaining arm did not receive neoadjuvant radiation and was treated only with the mFOLFOX6 regimen. The total radiation dose ranged from 46.0 to 50.4 Gy, with a daily dose of 1.8-2.0 Gy. After neoadjuvant treatment, all patients underwent radical surgery according to the standard of total mesorectal excision (TME).

### Study measurements and end points definition

The patients were partitioned into two groups based on the BMI cutoff value of 24, which is a well-established threshold for overweight with increasing metabolic risks in Chinese adults as defined by the Working Group on Obesity in China (WGOC).^19–21^ The definition of early-onset and late-onset groups was based on the cutoff of 50 years old as previously described.^4^^,^^22^ Tumor response grading (TRG) system was used to evaluate the pathologic response after neoadjuvant therapy. Patients with TRG 0 were defined as achieving pathological complete response (pCR), which means no viable residual tumor cells.^23^ The treatment-related complications included infection, bowel function impairment, anastomotic complication, and radiation impairment. The endpoints of this study consisted of 5-year overall survival (OS), disease-free survival (DFS), and locoregional recurrence (LRS). The prognostic markers for univariate Cox screening of OS, DFS, and LRS involved the neoadjuvant regimen, age, gender, mesorectal fascia (MRF), clinical TNM stage, tumor length, tumor thickness, distance to the anal verge (DTVA), and pretreatment carcinoembryonic antigen (pre-CEA).

### Statistical analysis

When comparing the demographic and clinicopathological characteristics, for continuous variables (presented as median and interquartile range), *t*-test was applied for normally distributed variables and otherwise Mann-Whitney U test was used. For categorical variables (presented as percentages), the chi-square test or Fisher exact test was used where appropriate, and the odds ratio (OR) was calculated by logistic regression. The Kaplan–Meier method with the log-rank test was used to analyze 5-year OS, DFS, and local recurrence. The hazard ratio (HR) and univariate screening for OS, DFS, and local recurrence were estimated by the Cox proportional hazards model. All statistical analyses were performed with R software (version 3.6.2, http://www.R-project.org), with a two-sided significance level of 0.05.

## Results

### Heterogeneity between rectal cancer patients with various BMI

A total of 244 patients with low BMI and 80 patients with high BMI with available prognosis data were enrolled in this study. Overall, the clinicopathologic characteristics of patients from two groups were similar, as shown in Table 1. The distribution of the neoadjuvant regimens was confirmed to be comparable between the low-BMI and high-BMI groups, consistent with the randomization scheme of the original FOWARC trial.

**Table 1.** Demographic and clinicopathological profiles of locally advanced rectal cancer patients in low-BMI and high-BMI populations.

	Low BMI (*N* = 244)	High BMI (*N* = 80)	Total (*N* = 324)	*P*
**Neoadjuvant radiation**				.714
** Without**	92 (37.7%)	32 (40.0%)	124 (38.3%)	
** With**	152 (62.3%)	48 (60.0%)	191 (62.0%)	
**Age median (IQR)**	56.00 (45.00, 64.25)	54.00 (47.00, 60.25)	55.00 (45.00, 63.00)	.791
**Gender**				.182
** Male**	172 (70.5%)	50 (62.5%)	222 (68.5%)	
** Female**	72 (29.5%)	30 (37.5%)	102 (31.5%)	
**cT**				.052
** 2**	2 (0.8%)	4 (5.0%)	6 (1.9%)	
** 3**	196 (80.3%)	63 (78.8%)	259 (79.9%)	
** 4**	46 (18.9%)	13 (16.2%)	59 (18.2%)	
**cN**				.385
** 0**	61 (25.0%)	14 (17.5%)	75 (23.1%)	
** 1**	98 (40.2%)	35 (43.8%)	133 (41.0%)	
** 2**	85 (34.8%)	31 (38.8%)	116 (35.8%)	
**cTNM**				.262
** II**	64 (26.2%)	16 (20.0%)	80 (24.7%)	
** III**	180 (73.8%)	64 (80.0%)	244 (75.3%)	
**MRF**				.692
** Negative**	165 (67.6%)	56 (70.0%)	221 (68.2%)	
** Positive**	79 (32.4%)	24 (30.0%)	103 (31.8%)	
**Tumor length median (IQR)**	4.20 (3.25, 5.10)	4.00 (3.00, 5.00)	4.00 (3.20, 5.00)	.242
**Tumor thickness median (IQR)**	15.00 (12.00, 20.00)	15.00 (12.00, 18.00)	15.00 (12.00, 19.00)	.237
**DTAV median (IQR)**	5.60 (3.77, 7.70)	5.15 (4.00, 7.00)	5.50 (3.80, 7.50)	.450
**Pre-CEA median (IQR)**	2.92 (1.55, 7.24)	2.50 (1.75, 6.78)	2.76 (1.57, 7.24)	.850

Abbreviations: DTAV, distance from the anal verge; IQR, interquartile range; pre-CEA, pretreatment carcinoembryonic antigen; MRF, mesorectal facia.

A univariate Cox screening was initially carried out to assess the heterogeneity of clinicopathological predictors for long-term survival outcomes in patients from non-obesity and obesity groups. In general, for markers such as neoadjuvant regimen, age, DTAV and MRF, their associations with survival outcomes differed between patients in low BMI and high BMI groups. A marker that had a positive correlation with survival in low-BMI patients might show a negative correlation in high-BMI patients, though some of these relationships were not significant (Figure 1). Notably, age had positive prognostic value for OS, DFS and LRS in low-BMI patients, while exhibited negative correlation with OS, DFS, and LRS in high-BMI patients, especially for LRS. Taken together, the prognostic marker profile was apparently heterogeneous between low-BMI and high-BMI patients.

**Figure 1.**
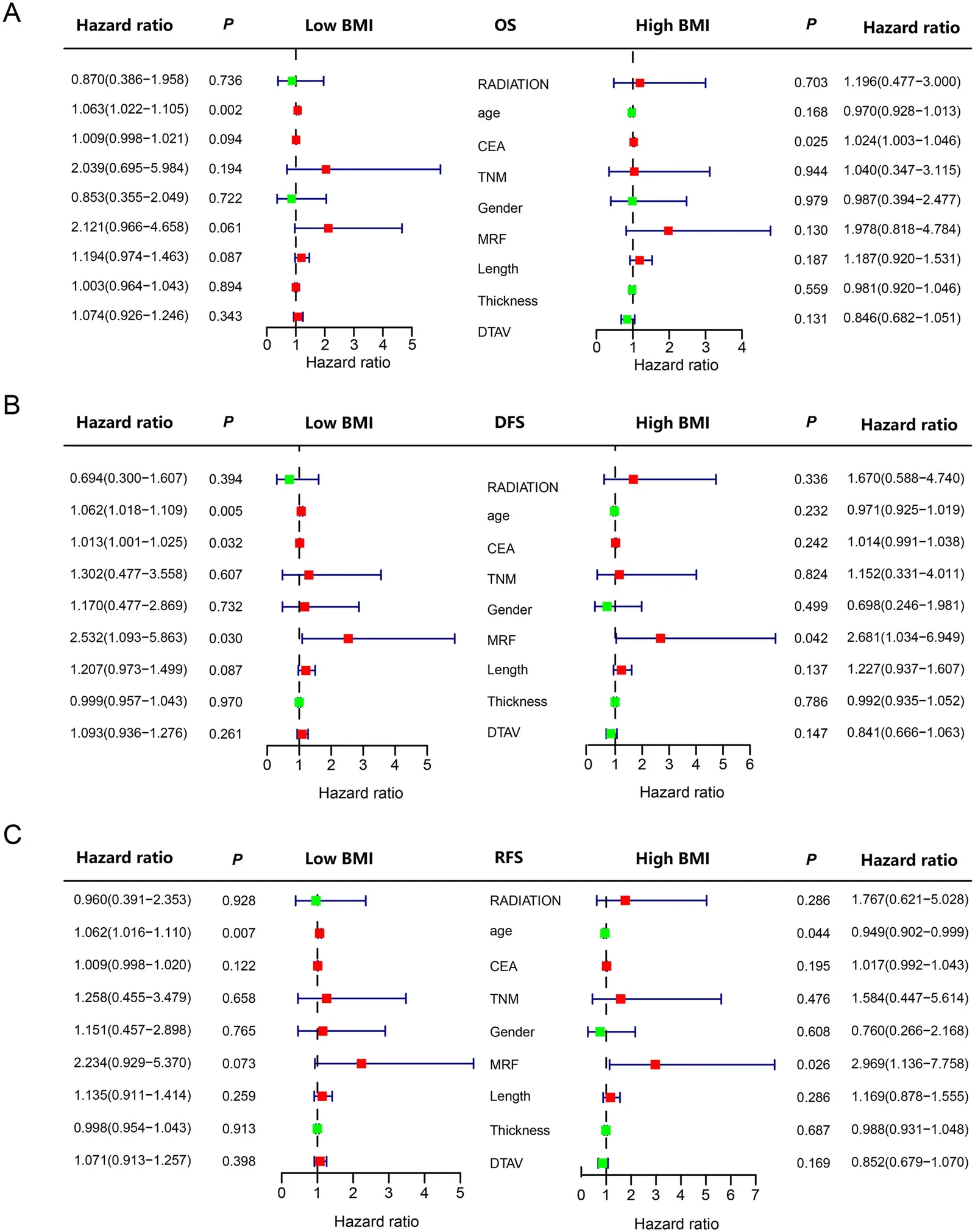
Heterogeneity of prognostic factors between rectal cancer patients with low and high BMI. Forest plots for univariate analysis of prognostic factors for long-term OS (A), DFS (B), and LRS (C) were shown in the low-BMI (left) and high-BMI (right) rectal cancer patients. DTAV, distance from the anal verge; MRF, mesorectal facia; pre-CEA, pre-treatment carcinoembryonic antigen.

### Prevalent aggressive phenotype in the early-onset rectal cancer with overweight and obesity

Subsequently, the clinicopathologic characteristics of patients with different BMI were compared. As shown in Figure 2A and D, the burden of aggressive phenotypes for rectal cancer (including T4 stage, MRF+, higher tumor length, and thickness) were slightly increased in low-BMI patients. As Figure 1 indicated that age might confound the risk impact of clinicopathologic markers, we further compared the burden of aggressive phenotypes among patients with early-onset or late-onset rectal cancers. Interestingly, for early-onset patients with high BMI, the proportion of aggressive phenotypes were increased, along with higher tumor length, tumor thickness and pre-operative CEA (Figure 2B and E). On the contrary, among late-onset patients, the aforementioned aggressive phenotypes wer/e all declined in high-BMI patients (Figure 2C and F). These contradictory trends further indicated the potential interaction between age and obesity.

**Figure 2.**
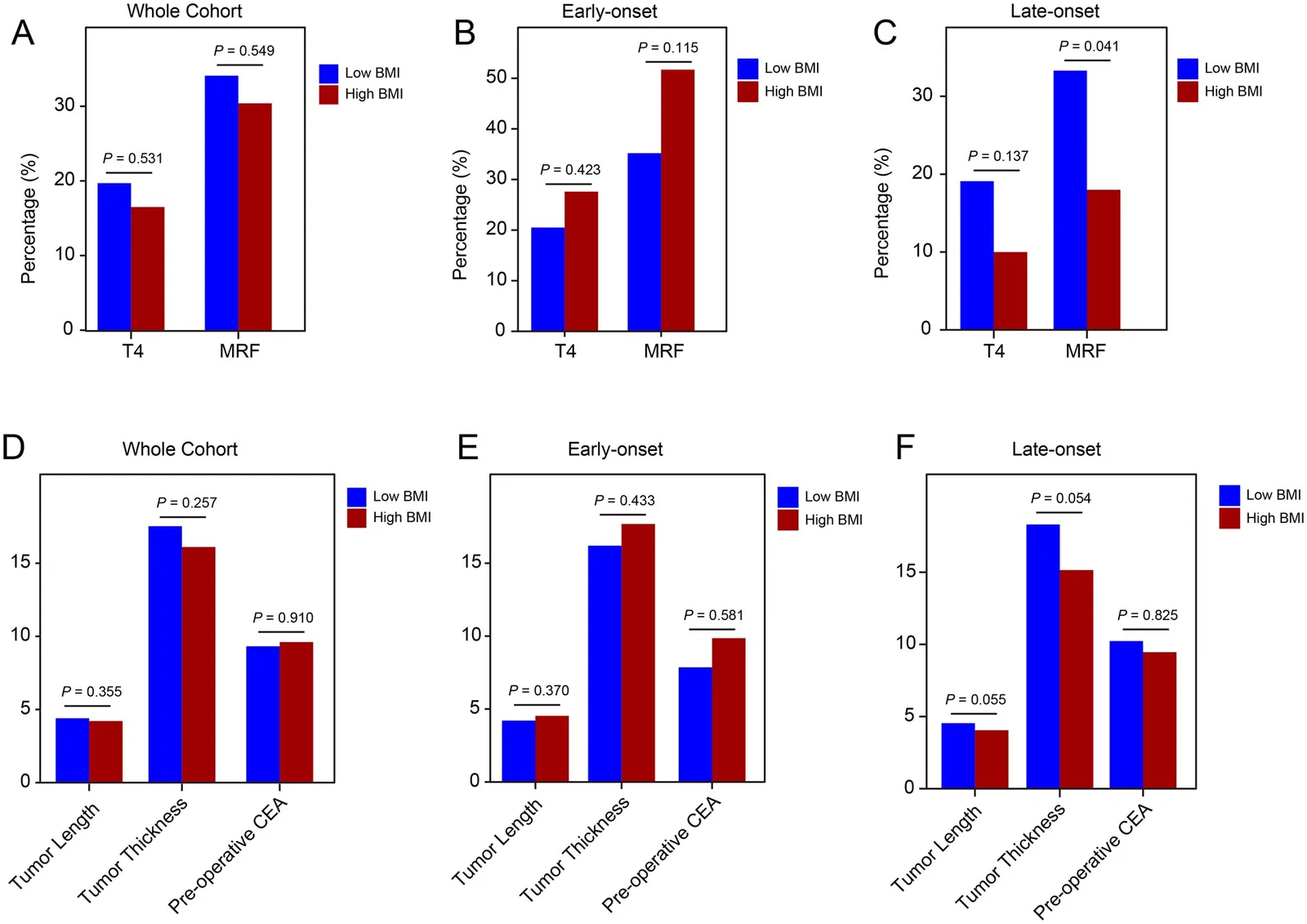
Distribution of aggressive clinical phenotypes in rectal cancer patients with different onset age and BMI. The proportion of T4 stage and MRF+ of low-BMI and high-BMI patients in whole cohort (A), early-onset (B), and late-onset (C) groups; the value of tumor length, tumor thickness and pre-CEA of low-BMI and high-BMI patients in whole cohort (D), early-onset (E), and late-onset (F) groups.

### Association of obesity and overweight with short-term outcome in young patients

Given the clinical profile heterogeneity, we subsequently assessed the short-term outcome following systemic regimen for low-BMI and high-BMI patients. For neoadjuvant treatment response, the proportions of patients achieved pathologic complete response (pCR) were slightly higher among low-BMI patients compared to those with high BMI (Figure 3A). To determine whether BMI imposed an additional risk of treatment-related complications, we compared the complication incidence between low-BMI and high-BMI patients. While not significant, the incidence of complications was lower in patients with low BMI than those with high BMI (Figure 3B). These implied that compared to nonobese patients, obesity might impair the tumor regression benefits from neoadjuvant treatment and increase the hazard of complications. The sub-group analysis was also performed for early-onset and late-onset rectal cancers, which showed that the pCR rates were largely consistent between low-BMI and high-BMI patients in both groups (Figure 3C and D).

**Figure 3.**
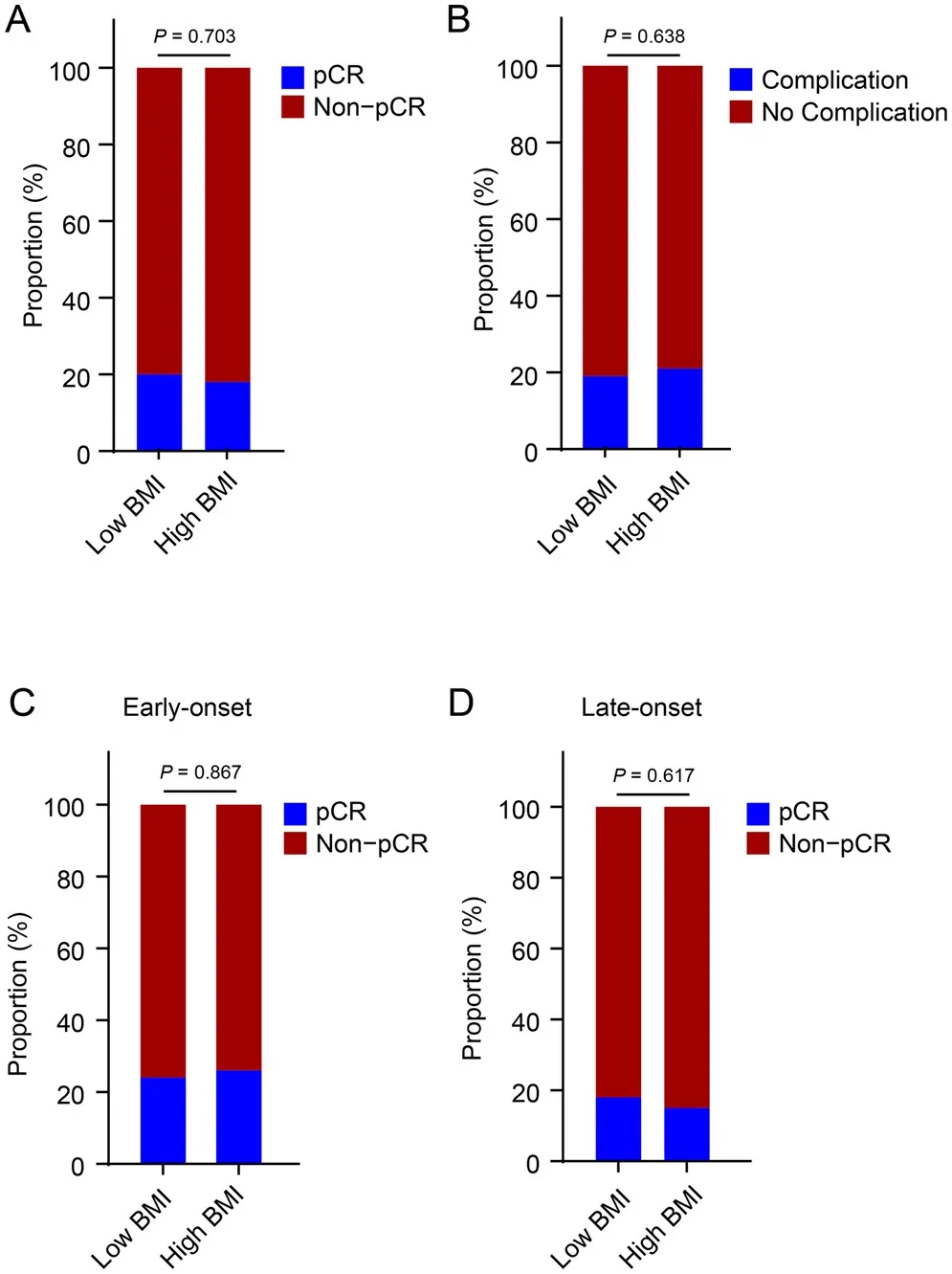
Short-term outcomes after neoadjuvant treatment in rectal cancer patients with low and high BMI. (A) The pathological complete response (pCR) rates after neoadjuvant treatment in low-BMI and high-BMI patients with rectal cancers. (B) The incidence of complications after neoadjuvant treatment in low-BMI and high-BMI patients with rectal cancers. (C) The pCR rates after neoadjuvant treatment of low-BMI and high-BMI patients in the early-onset group. (D) The pCR rates after neoadjuvant treatment of low-BMI and high-BMI patients in the late-onset group.

### Overweight and obesity contributed to worse long-term survival in early-onset rectal cancer patients

The long-term outcomes of patients from the low-BMI and high-BMI groups were further compared. The Kaplan-Meier analysis showed that the overall survival of low-BMI patients was significantly better than those with high BMI (Figure 4A). This discrepancy was also found in the analysis of disease-free survival (Figure 4D), which indicated the adverse effect of overweight on long-term prognosis for rectal cancer.

**Figure 4.**
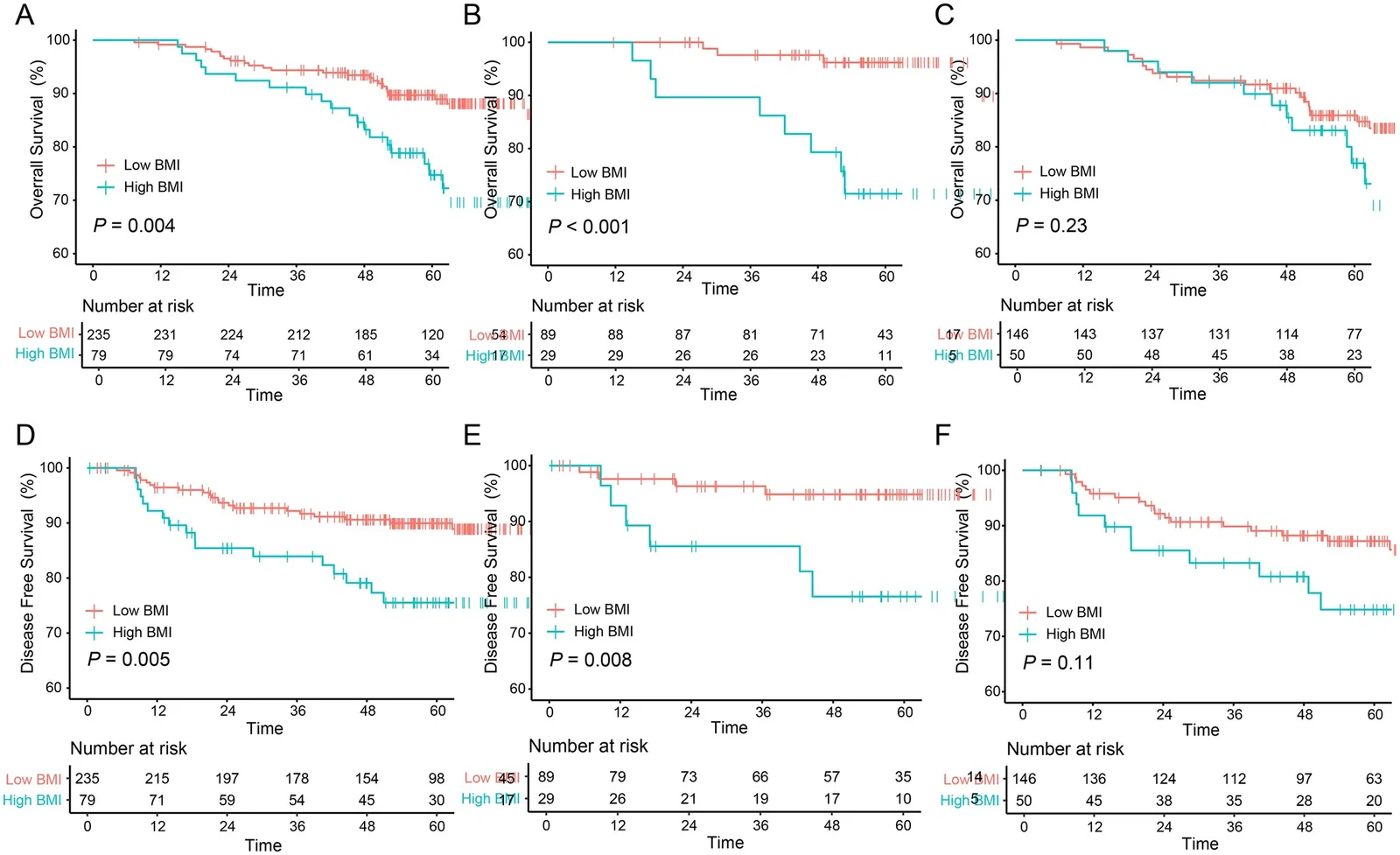
Long-term outcomes in rectal cancer patients with different onset age and BMI. K-M plots showing the long-term OS (A) and DFS (D) in low-BMI and high-BMI patients with rectal cancers. Sub-group analysis presented the OS (B, E) and DFS (C, F) for low-BMI and high-BMI patients with early-onset and late-onset rectal cancers.

As the univariate Cox screening in Figure 1 indicated that the prognostic value of age for long-term survival was heterogeneous between low-BMI and high-BMI patients, we performed a subgroup analysis regarding the age of onset. Of note, the results showed that the negative effect of elevated BMI was only found in early-onset patients (Figure 4B and E), but not in late-onset patients (Figure 4C and F). This suggested that early-onset patients might exhibit more vulnerability to the adverse effects of elevated BMI.

### Comparable outcomes between neoadjuvant chemotherapy and chemoradiotherapy in patients with elevated BMI

The FOWARC trial has previously demonstrated that neoadjuvant chemoradiotherapy did not confer significantly superior long-term survival compared with neoadjuvant chemotherapy alone in patients with locally advanced rectal cancer, a finding consistent with results from the PROSPECT trial.^18^^,^^24^ Considering the interaction of BMI with onset age and survival outcomes, we performed a subgroup analysis to explore the interference of overweight and obesity on the treatment outcomes of neoadjuvant radiotherapy. For short-term outcomes, the proportions of pCR were similar between low-BMI and high-BMI patients receiving neoadjuvant radiotherapy (Figure 5A). The risk of treatment-related complications did not increase for high-BMI patients compared to those with low BMI (Figure 5B). For long-term outcomes, addition of neoadjuvant radiation didn’t bring any survival benefits for low-BMI patients (Figure 5C and D), which was consistent with that in the high-BMI group (Figure 5D and F). Taken together, these exploratory analyses suggest that the addition of neoadjuvant radiation did not confer a statistically significant survival advantage in either BMI subgroup.

**Figure 5.**
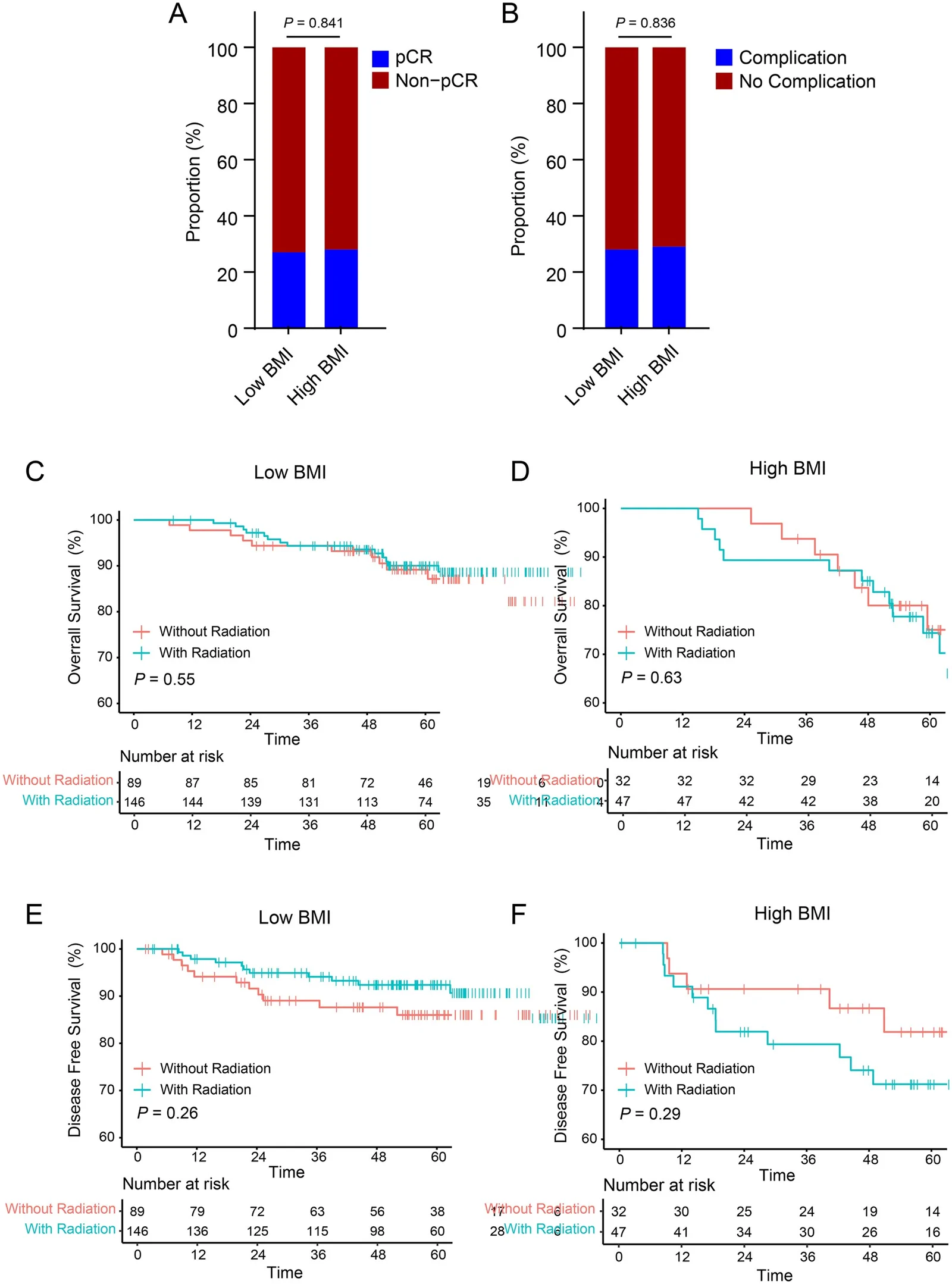
Short-term and long-term outcomes after neoadjuvant treatment with or without radiation in rectal cancer patients with high and low BMI. The pathological complete response (pCR) rates (A) and incidence of complications (B) after neoadjuvant treatment in low-BMI and high-BMI patients receiving neoadjuvant treatment with radiotherapy. The Kaplan–Meier analysis presented the overall (C, D) and disease-free (E, F) survival outcomes for patients receiving neoadjuvant treatment with or without radiation in low-BMI (C, E) and high-BMI (D, F) subgroups.

## Discussion

In this study, we found that the prognostic profile was heterogeneous between rectal cancer patients with high and low BMI, and age had opposing prognostic value in the two groups. Therefore, we investigated the interplay between overweight/obesity and treatment outcomes in rectal cancer patients, revealing that high BMI is associated with more aggressive tumor phenotypes and worse long-term survival specifically in the early-onset patients. Expectedly, the association of high BMI with poor survival outcomes also specifically in the early-onset patients. These findings indicated that elevated BMI is associated with aggressive tumor biology and significantly worse long-term survival exclusively in early-onset patients. In contrast, this deleterious association is entirely absent in the late-onset cohort. This age-dependent heterogeneity may provide a compelling explanation for the long-standing controversy surrounding the obesity paradox in colorectal cancer.

Our findings reflect fundamental biological differences in how adiposity interacts with host physiology across the lifespan. In the low-BMI group, increasing age with accompanying immunosenescence, comorbidities and accumulated genetic alteration may be the dominant driver of outcomes. Conversely, in the high-BMI group, the pro-tumorigenic effects of excess adiposity, including chronic low-grade inflammation, hyperinsulinemia, and altered adipokine signaling, may be especially pronounced in younger patients, whose more robust metabolic and immune systems are susceptible to obesity-induced dysregulation. This age-dependent vulnerability is further supported by epidemiological data showing that the association between obesity and cancer risk is stronger for EOCRC than LOCRC.^7^

The absence of a deleterious association between elevated BMI and survival in our late-onset group should not be interpreted as a protective effect of obesity. Rather, in older populations, the adverse metabolic consequences of excess adiposity may be obscured by several factors. First, BMI in older individuals may partially reflect preserved lean body mass rather than excess adiposity due to the high prevalence of age-related sarcopenia.^25^^,^^26^ Second, sarcopenic obesity is increasingly recognized as a distinct and adverse body composition phenotype that is paradoxically masked by BMI-based classification. The patients with sarcopenic obesity may have a normal or elevated BMI yet experience worse chemotherapy tolerance due to altered drug distribution in a reduced lean body compartment and poorer overall outcomes.^26^ Third, age-related immunosenescence and competing non-cancer mortality risks may overshadow obesity-specific inflammatory pathways in late-onset patients.^27^ In contrast, in the early-onset population, where sarcopenia is less prevalent, elevated BMI is more likely to reflect the unmasked biological consequences of excess adiposity and its associated metabolic dysregulation.^28^

The significant negative impact of overweight on long-term survival in EOCRC patients aligns with emerging evidences that obesity is a strong risk factor for EOCRC, and its increasing prevalence in younger generations is likely to substantially contribute to the increase in EOCRC incidence.^17^^,^^29^ Furthermore, emerging research suggests obesity promotes the carcinogenesis of EOCRC as well as its development and progression through mechanisms like metabolic syndrome and inflammation.^30^^,^^31^ Obesity fosters a pro-tumorigenic microenvironment characterized by chronic inflammation and metabolic dysregulation, which could contribute to the observed aggressive tumor biology and poorer outcomes in young, obese patients.^32^^,^^33^ The absence of significant BMI effects in late-onset patients parallels findings from the ACCENT database, where age-related immunosenescence may overshadow obesity-related pathways.^34^^,^^35^

These findings have immediate clinical implications for risk stratification. Young patients with rectal cancer and elevated BMI should be recognized as a distinct high-risk subgroup requiring intensified surveillance. In addition, weight control interventions may serve as a valuable adjuvant therapeutic strategy in EOCRC management. The American Society of Clinical Oncology (ASCO) guidelines recommend exercise, diet, and weight management during cancer treatment to improve outcomes related to quality of life and potentially treatment toxicity.^36^^,^^37^ For EOCRC patients, particularly those who are overweight, a structured weight management program implemented alongside or after primary treatment could mitigate the adverse effects of excess adiposity.^38^ Such programs could aim to modulate the systemic metabolic and inflammatory milieu, potentially enhancing treatment efficacy and improving long-term survival.^39^ The feasibility of investigating weight control in EOCRC patients should be considered in clinical trials. Our study highlights the urgent need for targeted weight management interventions for EOCRC patients, both during active treatment and in survivorship.

Beyond lifestyle modifications, pharmacologic interventions for weight management represent a promising strategy in oncologic care. Glucagon-like peptide-1 (GLP-1) receptor agonists, which promote significant weight loss, warrant investigation as potential adjuncts to cancer therapy, given preclinical data suggesting they may exert anti-tumor effects by attenuating obesity-associated inflammation and hyperinsulinemia.^17^ Additionally, nutritional interventions that modulate the gut microbiome, which is profoundly altered by both obesity and diet,^40^ could offer a dual-mechanism approach to simultaneously promote weight loss and foster a less pro-inflammatory gut environment.

It is important to contextualize our findings within the contemporary treatment landscape. The FOWARC trial predates the TNT era, which is now the established standard of care.^41^ Consequently, our exploratory observations regarding comparable outcomes between chemotherapy-alone and chemoradiotherapy approaches are derived from a pre-TNT framework and should not be interpreted as evidence supporting radiotherapy omission outside of clinical trials. Future studies in TNT cohorts are warranted to evaluate how BMI and body composition modify outcomes under contemporary treatment paradigms.

The conclusions of this study should be considered in the context of its design. First, while we identified a strong association, the causal role of obesity in driving aggressive EOCRC biology requires further mechanistic investigation. Second, BMI is an imperfect surrogate for adiposity and does not distinguish between lean and fat mass, and future prospective studies using CT-derived body composition analysis are essential to validate our findings and dissect the distinct roles of sarcopenia and visceral adiposity. Third, our analysis is based on a cohort from the pre-TNT era, and the applicability of our treatment-interaction findings to modern protocols needs to be established. Finally, the interaction analyses between BMI and treatment modalities were exploratory, which requires validation in larger independent cohorts.

## Conclusion

This study demonstrates a significant association of overweight and obesity with more aggressive tumor phenotypes and worse long-term survival in early-onset rectal cancer patients. This association was not observed in the late-onset cases. These findings highlight the vulnerability of early-onset patients with high BMI, which highlights the need for BMI-specific therapeutic strategies and weight management interventions in these vulnerable cases.

## Data Availability

The datasets used and/or analyzed during the current study are available from the corresponding author upon reasonable request.
